# A FUNCTIONAL EPIGENETIC CLOCK FOR RATS

**DOI:** 10.1093/geroni/igz038.128

**Published:** 2019-11-08

**Authors:** Morgan E Levine, Ross McDevitt, Luigi Ferrucci, Rafael deCabo

**Affiliations:** 1 Yale University School of Medicine, New Haven, Connecticut, United States; 2 National Institute on Aging, Baltimore, Maryland, United States

## Abstract

Evidence from humans suggests that incorporation of phenotypic aging measures in the development of epigenetic clocks leads to more functionally relevant biomarkers. As a result, the aim of this study was to utilize a deeply phenotyped rat cohort—that included data from rotarod, open field, frailty index, and FACS—to generate a novel epigenetic clock. DNA methylation was assessed via reduced representation bisulfite sequencing (RRBS) for n=142 male Fischer rats from NIA aging colony, ranging in age from 1 to 27 months. Phenotypic traits were combined to generate an multi-system aging measure that was then used to train the epigenetic clock. Using an independent validation sample, age-adjusted epigenetic clock measures were associated with numerous traits, including: open field time resting (p=0.005), open field time climbing (p=0.001), body weight (p=0.02), and rotarod max (p=0.04). In moving forward, it will be important to examine cross-species comparisons, longitudinal change, and functional enrichment.

